# A Retrospective Analysis of Robertsonian Translocations from a Single Center in China

**DOI:** 10.1007/s43032-023-01398-3

**Published:** 2023-11-06

**Authors:** Wan Lu, Jihui Zhou, Huihua Rao, Huizhen Yuan, Shuhui Huang, Yanqiu Liu, Bicheng Yang

**Affiliations:** Medical Genetic Center, Jiangxi Key Laboratory of Birth Defect Prevention and Control, Jiangxi Maternal and Child Health Hospital, Nanchang, Jiangxi China

**Keywords:** Robertsonian translocations, Chromosomal abnormality, Prenatal diagnosis, Assisted reproductive technology

## Abstract

Robertsonian translocations (ROBs) are the most common structural chromosomal abnormalities in the general population, with an estimated incidence rate of 1/1000 births. In this study, we retrospectively analyzed the cases of ROBs from September 2015 to August 2022 and totally identified ROB carriers from 84,569 specimens karyotyped in a single accredited laboratory in China, including 189 cases of balanced ROBs and 3 of mosaic ROBs. Microsoft Excel and descriptive statistics were used to record and analyze the collected data. The male/female ratio of ROBs is 1/1.29, with der(13;14) and der(14;21) being the main karyotypes. Among the 192 patients, 7 were lost to follow-up, 82 had given birth, and 103 were childless (such as miscarriage, fetal chromosomal abnormalities, in vitro fertilization (IVF) failure, or divorce). A total of 44 amniocenteses were performed in 42 couples; ROB cases with natural pregnancies showed that the normal karyotype and balanced ROBs of fetal accounted for 66.67% (16/24), while the results of assisted pregnancies showed 90.00% (18/20). This study represents the largest collections of ROBs in Jiangxi population and reminder that the ROB carriers can achieve the ideal outcome for pregnancy with the appropriate genetic guidance and assisted reproductive technologies (ART).

## Introduction

Robertsonian translocations (ROBs) are special forms of translocation in which the entire long arm of proximal centromere chromosomes is fused, also known as centromere fusion [1]. Translocations can occur between homologous chromosomes, but are more common between non-homologous pairs. Acrocentric chromosomes (13, 14, 15, 21, 22) can fuse to form 5 homologous ROBs and 10 heterologous ROBs; involving translocations of chromosomes 13 and 14 [der(13;14)] is the most common rearrangement. The majority of heterologous ROBs are inherited from one partner carrier, the rest are produced in the meiosis I of oogenesis. Almost all homologous ROBs are formed during mitosis. Although ROBs may have a normal phenotype and almost no deletion of genetic material, their pregnancies are at an increased risk of infertility and miscarriage and may lead to a livebirth with intellectual disability secondary to unbalanced chromosomal arrangement [2, 3]. It was reported that the incidence of ROBs is 0.1% in the general population, 1.1% in patients with recurrent pregnancy loss, and 3% in infertile men, making ROB rearrangement one of the most common structural chromosomal abnormalities [4].

Nearly one million newborns are born in China each year with different types of birth defects. Chromosome abnormalities, including ROBs, are considered to be one of the most important causes [5]. Chromosomal abnormalities refer to genetic diseases caused by changes in chromosome structure and (or) abnormal number and are important causes of infertility, fetal abortion, neonatal malformation, mental retardation, developmental delay, and other diseases. Early and accurate detection is an important guarantee for the diagnosis of chromosomal abnormalities, which is of great significance for preventing the occurrence of abortion and improving the rate of good birth; hence, it is valuable to establish a birth defect surveillance system. However, only a few studies have reported data involving ROBs in Chinese populations [6, 7]. In order to know the occurrence of ROBs and pregnancy outcomes in Jiangxi province of China and to provide reference for genetic counseling and fertility guidance, 192 cases of ROBs were collected and tracked in this study.

## Materials and Methods

A 7-year retrospective study from September 2015 to August 2022 was carried out in couples with a clinical diagnosis of infertility, miscarriage, recurrent abortion, and genetic counseling at Jiangxi Key Laboratory of Birth Defect Prevention and Control of Jiangxi Maternal and Child Health Hospital. The age of the patients ranged from 20 to 52 years old. The reproductive histories of each patient were recorded in detail, and peripheral blood was taken. Inform consent was signed before the study.

Peripheral blood karyotype: 2 mL of peripheral blood was taken with heparin anticoagulation. About 0.5 mL of blood was inoculated into lymphocyte culture medium (Dahui Biotechnology) at 37 ℃ for 72 h. Four hours before the end of culture, colchicine (20 μg/mL) was added and prepared by conventional method. Cytogenetic analysis was performed at approximately 400-band level. Leica Cytovision System was used to analyze 5 karyotypes and count 30 karyotypes and at least 50 karyotypes in chimeric cases. Karyotypes were named according to the International System for Human Cytogenetic Nomenclature (ISCN) 2020.

Fetal karyotype: Chromosome abnormality was one of the important indications of cytogenetic prenatal diagnosis [8]. Informed consent forms were signed by pregnant women willing to undergo amniocentesis. Amniotic fluid samples were collected by ultrasound-guided transabdominal amniocentesis. Cells were cultured and prepared for G-banding karyotyping using standard protocols. Leica karyotype analysis system was used to count 20 karyotypes and analyze 5 karyotypes. Karyotypes were named according to ISCN 2020.

## Results

### Clinical Features of ROB Carriers

Among the 84,569 specimens karyotyped in this study, 192 cases were identified as ROB carriers. According to the medical records, the main reasons for treatment of ROBs were infertility and miscarriage, accounting for 81.25% of 156 cases, including 6 cases of azoospermia. The rest cases of ROBs were confirmed with the situations like pregnancy examination or genetic counseling, had unhealthy children, such as chromosomal abnormalities and congenital malformations, abnormal fetal ultrasound or high risk of non-invasive prenatal testing (NIPT) during pregnancy, and menstrual disorder (Table 1). Based on the miscarriage history, most cases had at least two spontaneous abortions (Table 2).

**Table 1:** Reasons for treatment of ROBs

Reasons for treatment	Cases	Proportion (%)
Primary infertility	65	33.85
Secondary infertility	10	5.21
Miscarriage^#^	81	42.19
Physical examination/genetic counseling	15	7.81
Having unhealthy children	11	5.73
Abnormal fetal ultrasound structure or NIPT high risk during pregnancy	7	3.64
Menstrual disorder	3	1.56
Total	192	100.00

Note: ^#^6 cases with a history of both miscarriage and abnormal delivery were included in the miscarriage category. *NIPT*, non-invasive prenatal testing

**Table 2:** Distribution based on number of miscarriages

No. of miscarriages	Total (*n*)	Proportion (%)
1	22	27.16
2	38	46.91
3	16	19.75
≥ 4	5	6.17
Total	81	100.00

### Karyotype Distribution of ROBs

The 192 ROB cases can be classified into 189 balanced ROBs and 3 mosaic ROBs, 84 males and 108 females, producing male-to-female ratio of 1:1.29. There were 9 of homologous [der(13;13), der(21;21) and der(22;22)] and 183 of nonhomologous ROBs, while der(13;14) was the most common karyotype accounting for 60.94% (117/192). The detailed karyotype distribution of ROBs is shown in Table 3.

**Table 3:** Karyotype distribution of ROBs

Karyotype distribution	Male	Female	Cases	Proportion (%)
der(13;13)	1	3	4	2.08
der(13;14)	57	60	117	60.94
der(13;15)	2	0	2	1.04
der(13;21)	2	2	4	2.08
der(13;22)	0	1	1	0.52
der(14;15)	4	4	8	4.17
der(14;21)	11	17	28	14.58
der(14;22)	1	4	5	2.60
der(15;21)	0	2	2	1.04
der(15;22)	3	4	7	3.65
der(21;21)	0	2	2	1.04
der(21;22)	0	4	4	2.08
der(22;22)	1	2	3	1.56
45,X,del(X)(q26),der(14;15)	1	0	1	0.52
46,XXY,der(13;14)	0	1	1	0.52
45,XX,der(14;22)/46,XX	0	0	1	0.52
45,XX,der(15;21)/46,XX	0	1	1	0.52
45,XY,der(21;21)/46,XY	1	0	1	0.52
Total	84	108	192	100

### Pregnancy Outcomes of ROB Carriers

The 192 ROBs were followed up by telephone in March 2023, and the loss rate was 3.65% (7/192). Among the 185 tracked cases, 82 had given birth, 90 were childless (including infertile, miscarriage, abnormal fetal chromosome, and in vitro fertilization failed), 6 were divorced, 5 are ongoing pregnant, and 2 cases had adopted a child. There were 342 gravidities in total among the ROBs cases; although 170 pregnancies ended in miscarriage, at least 43.27% (148/342) were pregnant with normal phenotype fetus (Table 4).

**Table 4:** Detail pregnancies distribution of ROBs

	No. of pregnancies	Proportion (%)
History of 1 miscarriage	22 (22 cases)	6.43
History of 2 miscarriages	76 (38 cases)	22.22
History of 3 miscarriages	48 (16 cases)	14.04
History of 4 miscarriages	12 (3 cases)	3.51
History of 6 miscarriages	12 (2 case)	3.51
Normal live born children^#1^	148	43.27
Abnormal karyotypes^#2^	15	4.39
Ongoing pregnancies	5	1.46
Others^#3^	4	1.17
Total	342	100.00

Note: ^#1^normal or balanced karyotype with normal phenotype; ^#2^trisomy or sex chromosome abnormality; ^#3^the child has dead without any test

### Prenatal Diagnosis of ROB Carriers

Forty-two pregnant ROB cases, including 24 natural pregnancies and 20 assisted pregnancies, have accepted 44 amniocenteses to test the fetal karyotypes. The results of prenatal diagnosis in ROBs cases with natural pregnancies showed that the normal karyotype and balanced ROBs of fetal accounted for 66.67% (16/24) and trisomy and other abnormities accounted for 33.33% (8/24), while the results of assisted pregnancies showed that 90.00% (18/20) were normal karyotype and balanced ROBs, and the other 2 cases were Trisomy-21 Syndrome (Table 5).

**Table 5:** Amniocentesis in 42 ROB carriers

Number	Karyotypes	Fetal karyotypes
Natural pregnancy		
1^#1^	der(13;21)	45,XN,der(13;21)mat
2	der(13;14)	46,XN
3	der(14;21)	45,XN,der(14;21)pat
4	der(15;21)	45,XN,der(15;21)mat
5	der(14;21)	45,XN,der(14;21)pat
6	der(14;21)	47,XN, + 21
7^#2^	der(13;14)	47,XN, + 13
8	der(13;14)	45,XN,der(13;14)pat
9	der(14;21)	46,XN
10	der(14;22)	45,XN,der(14;22)mat
11	der(14;21)	45,XN,der(14;21)mat
12	der(13;14)	47,XXY
13	der(14;22)	46,XXY,der(14;22)mat
14	der(21;21)	46,X,inv(Y),der(21;21)mat
15	der(14;21)	46,XN
16	der(13;14)	46,XN
17	der(21;22)	45,XN,der(21;22)mat
18	45,X,del(X)(q26),der(14;15)	46,X,del(X)(q26)mat
19	der(13;21)	47,XN, + 21
20	der(21;22)	47,XN, + 21
21	der(13;14)	46,XN
22	der(14;15)	45,XN,der(14;15)mat
23	der(13;14)	46,XN
24	der(13;14)	46,XN
Assisted pregnancy		
1	der(14;21)	46,XN
2	der(13;14)	46,XN
3^#1^	der(13;21)	46,XN
4	der(13;14)	46,XN
5^#2^	der(13;14)	46,XN
6	der(14;21)	47,XN, + 21
7	der(13;14)	45,XN,der(13;14)mat
8	der(13;14)	46,XN
9	der(13;14)	46,XN
10	der(14;21)	46,XN
11	der(13;14)	46,XN
12	der(13;14)	45,XN,der(13;14)mat
13	der(13;14)	45,XN,der(13;14)mat
14	der(13;14)	46,XN
15	der(14;22)	45,XN,der(14;22)mat
16	der(15;22)	46,XN
17	der(13;14)	46,XN
18	der(13;14)	46,XN
19	der(14;21)	47,XN, + 21
20	der(14;21)	45,XN,der(14;21)mat

Note: ^#1^number 1 of natural pregnancy and number 3 of assisted pregnancy are from same case; ^#2^number 7 of natural pregnancy and number 5 of assisted pregnancy are from same case

## Discussion

Robertsonian translocation (ROB) is a common chromosomal abnormality, which is formed by the fusion of two acrocentric chromosomes. It is related to the oligospermia in male adults, miscarriage or infertility in female adults, and occurring at a rate of about 1/1000 newborns [1, 5]. Although the number of chromosomes was reduced, the main genetic material was not lost so that the ROB carriers were usually phenotypically normal except for reproductive disorders. In the process of meiosis of ROB carriers, apart from a normal gamete and a ROB, approximately two-thirds of their gametes feature disomy or nullisomy, that easily lead to early embryo abortion and stillbirth, means almost one-third of the probability to give birth to a phenotypically normal child [9, 10]. In our study, 81.25% of ROB carriers came to the clinic due to infertility or miscarriage, and 59 of them had at least two spontaneous abortions. A total of 342 pregnancies were recorded in the 185 tracked cases, of which 49.71% (170/342) ended in abortion and 43.27% (148/342) were born with normal phenotype, higher than the theoretical value. Therefore, history of multiple miscarriages is an obvious indication for genetic diagnosis.

Individuals with ROBs usually have only 45 chromosomes, because the short arms of two acrocentric chromosomes are lost during subsequent cell divisions [11, 12]. The most common type of ROB occurs between chromosomes 13 and 14, and the second common is between chromosomes 14 and 21; other possible combinations are infrequent [13]. Consistent with previous researches, among the 192 cases in our study, der(13;14) was the most common karyotype, accounting for 60.94%, followed by der(14; 21), with a total of 28 cases accounting for 14.58% (28/192). The gender of ROBs can obviously affect the proportion of meiotic segregation patterns. Studies of chromosomal segregation in ROBs have shown that the male carriers had a significantly higher proportion of normal/balanced gametes than the female carriers [14, 15]. In our study, we found 192 ROBs in the 84,569 patients, with a male to female ratio of 1:1.29. This proportion was contrary to previous studies, which may be attributed to the traditional belief that more miscarriages were caused by female factors than by male ones, so more women in this region coming for pregnancy counseling [16].

In recent decades, great progress in assisted reproductive technology (ART), encompass in vitro fertilization (IVF) with or without intracytoplasmic sperm injection (ICSI), have enabled previously untreatable infertility to be successfully treated [17, 18]. ART has become an important method to effectively improve the clinical pregnancy rate for the infertile couples. However, the patients undergoing ART still have the risk of genetic disorders, such as embryonic chromosomal abnormality. Researches have showed that, 20% of pregnant women receiving ART had spontaneous miscarriage in early stage of pregnancy [19, 20]. Therefore, it is necessary to have proper counseling and testing before preparing for pregnancy, especially for the embryo implantation in ART. Preimplantation genetic diagnosis (PGD) is a genetic analysis of an embryo or gamete, and embryos without chromosome or genetic abnormality are selected to transplanted into the mother’s uterus [21]. Therefore, for high-risk couples, the genetic material of embryos can be analyzed before embryo transfer to diagnose whether there are abnormalities and select healthy embryos for transplantation. For female carriers, PGD can be used to screen abnormal polar bodies, while male carriers need to use ICSI and PGD to select normal or balanced translocation embryos. It is proved that PGD can obviously reduce the abortion rate of ROBs carriers [22, 23]. Although guidelines recommend that couples with chromosome abnormalities could undergo amniocentesis to test fetal chromosome, amniocentesis was an invasive procedure; considering the risk of pregnancy loss or preterm birth, 42 women underwent a total of 44 amniocenteses during the second trimester of pregnancy. The proportion of normal karyotype and ROB in natural and ART pregnancies was 66.67% and 90%, respectively. The application of ART and PGD can remarkably improve the probability to have phenotypically normal children for ROB carriers.

This study provides data reference for pregnancy in Robertsonian translocation carriers. However, it has certain limitations, some carriers from diagnosis to follow-up only half a year; the next step will be to regularly follow up their pregnancy. Secondly, there is a lack of records of newborns after birth. We will follow up the growth and development of children with Robertson carriers. Thirdly, the portion of prenatal diagnosis is relatively low; concern about the pregnancy loss caused by amniocentesis is the main reason, especially for couples with IVF. Although prenatal diagnosis is based on voluntary principles, the probability of Robertson translocation carriers giving birth to trisomy and other abnormalities is significantly higher than that of other couples, and we will further strengthen the publicity and education of prenatal diagnosis.

To sum up, people with the normal type may carry balanced translocations or inversions that generally have no effect except for fertility problems. Parents with infertility and poor pregnancy history are recommended to undergo peripheral karyotyping to detect any balanced structural chromosomal abnormality. Couples with chromosomal abnormality should be given proper fertility guidance. Although PGD could reduce the pregnancy loss, the ability to achieve successful natural pregnancy should be carefully considered, especially for young fertile couples without a history of recurrent miscarriage [24]. Couples with chromosomal abnormalities, advanced age, and a history of adverse pregnancy could choose PGD. Preimplantation genetic diagnosis could be used before embryo implantation, and prenatal diagnosis (chorion villus sampling, amniocentesis, and percutaneous umbilical blood sampling) could be performed when necessary. More importantly, couples are advised to undergo genetic testing before pregnancy, which can prevent miscarriage or fetal abnormalities caused by chromosomal diseases or monogenic diseases.

## Data Availability

Not applicable.
